# Extracellular superoxide dismutase, a molecular transducer of health benefits of exercise

**DOI:** 10.1016/j.redox.2020.101508

**Published:** 2020-03-19

**Authors:** Zhen Yan, Hannah R. Spaulding

**Affiliations:** a Center for Skeletal Muscle Research at Robert M. Berne Cardiovascular Research Center, University of Virginia School of Medicine, Charlottesville, VA, 22908, USA; b Department of Medicine, University of Virginia School of Medicine, Charlottesville, VA, 22908, USA; c Department of Pharmacology, University of Virginia School of Medicine, Charlottesville, VA, 22908, USA; d Department of Molecular Physiology and Biological Physics, University of Virginia School of Medicine, Charlottesville, VA, 22908, USA

**Keywords:** EcSOD, Exercise, Oxidative stress, Endothelial dysfunction, Endothelial cell activation

## Abstract

Extracellular superoxide dismutase (EcSOD) is the only extracellular scavenger of superoxide anion (O_2_^.-^) with unique binding capacity to cell surface and extracellular matrix through its heparin-binding domain. Enhanced EcSOD activity prevents oxidative stress and damage, which are fundamental in a variety of disease pathologies. In this review we will discuss the findings in humans and animal studies supporting the benefits of EcSOD induced by exercise training in reducing oxidative stress in various tissues. In particularly, we will highlight the importance of skeletal muscle EcSOD, which is induced by endurance exercise and redistributed through the circulation to the peripheral tissues, as a molecular transducer of exercise training to confer protection against oxidative stress and damage in various disease conditions.

## 1 Introduction

Oxygen is indispensable for life; however, under certain situations it has deleterious effects due to the formation and activity of a number of chemical compounds, known as free radicals or reactive oxygen species (ROS) [46]. Fortunately, cellular enzymatic and nonenzymatic mechanisms are in place to neutralize ROS. Among the members of the enzymatic defense system, superoxide dismutases (SODs) are an ubiquitous family of enzymes that catalyze the dismutation of superoxide anions (O_2_^.-^) as the first line of defense against ROS [117]. SODs reduce O_2_^.-^ to oxygen and hydrogen peroxide (H_2_O_2_), and H_2_O_2_ is further neutralized to water through enzymatic reactions by catalase (CAT) or glutathione peroxidase (GPX). These reactions are necessary to maintain redox balance. However, excessive ROS production can exceed the capacity of the antioxidant defense system leading to oxidative stress, which causes cellular damage by oxidizing proteins, lipids, DNA/RNA, and other macromolecules. Oxidative stress has been shown to play an important role in many disease pathologies, such as cachexia, sepsis, hypertension, myocardial infarction (MI), chronic heart failure (CHF), chronic obstructive pulmonary disease (COPD), acute respiratory distress syndrome (ARDS) and multiple organ dysfunction syndrome (MODS) [31,35]. There are three SOD isoenzymes; each resides in a distinct cellular location SOD1, or CuZnSOD, is a copper and zinc-containing homodimer in cytoplasm. SOD2, or MnSOD, exists as a tetrameric manganese-containing enzyme in the matrix of mitochondria. SOD3, or EC-SOD, is a copper and zinc-containing tetrameric enzyme that is secreted from the producing cells into the extracellular space [117].

EcSOD is widely expressed in many tissues/organs with the highest levels in the lung and kidney [30,72,117]. EcSOD is so far the only known antioxidant enzyme that functions to scavenge biologically toxic O_2_^.-^ in the extracellular space. EcSOD is produced within cells, then processed and secreted. Immature EcSOD protein has an N-terminus with targeting signal that instructs its secretion to the extracellular space. Once this targeting signal is cleaved, the mature form gains enhanced protein stability due to a glycosylation of asparagine at position 89 [80]. EcSOD contains an activity site where copper and zinc ions bind, which is essential for ROS neutralizing function. Finally, EcSOD has a heparin binding domain (HBD) on the C-terminus, consisting of six positively charged lysine and arginine residues. This HBD is responsible for EcSOD binding to proteoglycans, such as heparin, collagen and fibulin, which allows for EcSOD localization to the cell surface and extracellular matrix. In order for EcSOD to be released into plasma and other fluids, carboxylases and furin-like proteases have to cleave EcSOD near the HBD. In this manner, EcSOD acts not only on the cell surface and in the extracellular matrix of producing cells in a paracrine manner but can also be distributed systemically to other tissues in an endocrine manner. *In vivo* evidence support that EcSOD enriches at endothelial cells in producing cells as well as the targeted peripheral tissues [11,12,78]. Detoxification of vascular O_2_^.-^ by EcSOD preserves NO bioavailability [54], which is critical for normal vasoreactivity. Furthermore, EcSOD can be endocytosed into the targeted endothelial cells through clathrin-mediated pathway depending on HBD. The endocytosed EcSOD does not translocate to the nucleus but may function as antioxidant intracellularly in the targeted cells [18]. Finally, a common human single nucleotide polymorphism (SNP) of EcSOD with a substitution of arginine at 213 with glycine (R213G) in HBD in about 5% of the population, which lead to impaired binding to cell surface and extracellular matrix, does not affect the enzymatic activity but lead to impaired antioxidant function in various model systems [2,16,18,56,79].

The functional importance of EcSOD in humans has been deduced from reduced EcSOD expression a variety of chronic diseases [15,22,63,95,102]. Genetic evidence also supports a causal role of reduced EcSOD activity in chronic disease pathologies [25]. For example, EcSOD R213G SNP has been associated with increased risk or poor prognosis of oxidative stress-related diseases, such as acute lung injury, ischemic heart diseases, and kidney failure [7,56,59,111]. The role of HBD in EcSOD distribution in the tissue reveals the importance of targeting specificity for EcSOD to be an effective extracellular antioxidant. For example, a T-allele of rs2284659 variant in the promoter of the EcSOD gene is associated with higher plasma EcSOD and lower plasma advanced oxidation protein products. This gene variant is inversely associated with incidence of MI and all-cause of mortality in people with type 1 and type 2 diabetes [74], suggesting that EcSOD plays an important role in protection against diabetes-related oxidative stress.

Experimental studies in animal models show that genetic manipulation with deletion or overexpression of the *EcSOD* gene exacerbates or prevents chronic disease states, providing evidence for a causal role of reduced ectopic expression of EcSOD in disease development [13,41,50,66,69,70,89,105,114]. For example, *EcSOD* knockout mice display cardiac fibrosis and ventricular hypertrophy, suggesting a role of normal expression of EcSOD in deterring these processes [105]. Furthermore, a novel rat strain with a missense mutation that alters a single amino acid (E124D) of EcSOD that produces a malfunctioning protein (*EcSOD*^*E124D*^) in the Dahl/Salt Sensitive (Dahl/SS) background developed age-dependent arterial hypertension, kidney failure and cardiac hypertrophy, suggesting that endogenous EcSOD protects multiple organ systems against extracellular oxidative stress [45]. On the contrary, ectopic overexpression of EcSOD mediated by adeno-associated virus (AAV) provided significant protection against ischemic damage in skeletal muscle and the heart [60,93], indicating enhanced EcSOD expression is sufficient for protection against oxidative damage. Finally, reduced EcSOD abundance and vascular dysfunction in aged rats could be corrected by adenovirus-mediated gene transfer of EcSOD, which appear to be dependent on the HBD [9].

**Fig. 1:**
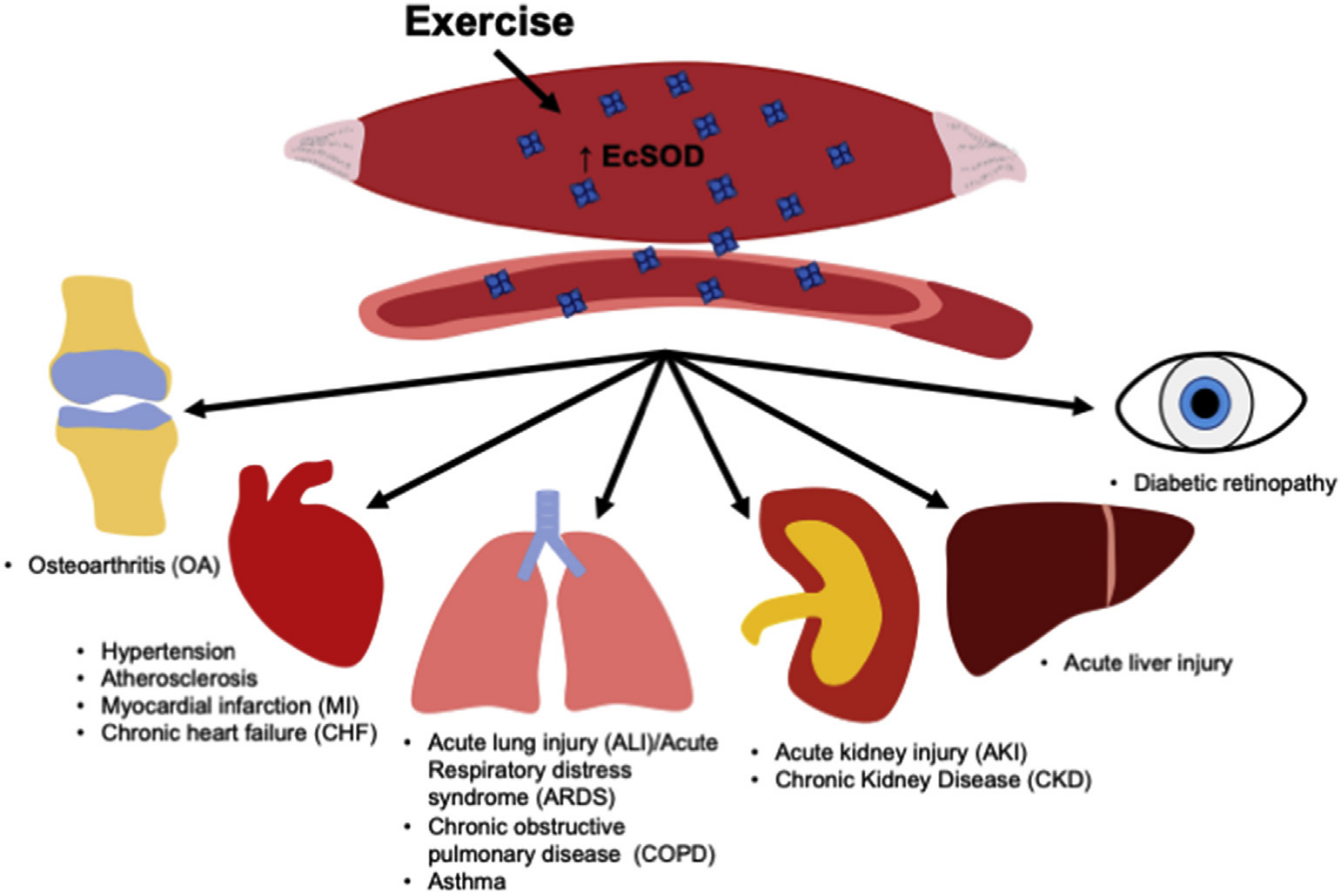
**Enhanced skeletal muscle EcSOD expression by exercise promotes mitigation of oxidative stress and damage in a variety of peripheral tissues and disease conditions.** Accumulating evidence supports that reduced EcSOD abundance and activities in tissues are associated with many disease conditions, and increased EcSOD activity is protective against oxidative stress and damage under these disease pathologies. Endurance exercise increases EcSOD abundance in skeletal tissue, which can be redistributed to peripheral tissues via the circulation to combat ROS and oxidative damage.

Overall, EcSOD has recently emerged as a promising therapy for protection of vital tissues/organs under various disease conditions related to oxidative stress. Recent findings suggest that endurance exercise promotes EcSOD expression in skeletal muscle, the largest organ in our body, leading to elevated levels of EcSOD in other peripheral organs. This humoral function of EcSOD induced by exercise training may become effective therapeutics for many disease conditions (Fig. 1). Several landmark reviews have provided elegant overview of the regulation and function of EcSOD in health and disease [25,34,117]. In this review, we will focus on the benefits of EcSOD and how exercise may increase skeletal muscle EcSOD to protect vital tissues/organs against oxidative damage.

## 2 Exercise and EcSOD

The antioxidant capacity of mammalian organ systems matches very well with the rates of oxygen consumption and free radical production. Oxidative tissues, such as skeletal muscle, heart and kidney, have the greatest antioxidant enzyme activity. Consistent with the fact that acute exercise, particularly intense or prolonged exercise, increases free radical production and that reactive free radicals are often counterbalanced by antioxidant defense systems under the control of signaling pathways in response to increased free radicals to maintain homoeostasis, exercise training or physical activity has been associated with reduced oxidative stress in many studies in humans. Indeed, controlled clinical trials show that regardless of intensity, volume, type of exercise, and studied population, the antioxidant indicators tend to increase, and pro-oxidant indicators tend to decrease after exercise training [23].

Reduction of oxidative stress in organ systems by exercise training is likely due to enhanced antioxidant defenses in tissues [88]. For example, slow-twitch, oxidative fibers in skeletal muscle with high oxidative capacities have higher antioxidant capacities compared with fast-twitch, glycolytic fibers with lower oxidative potential. While majority of the effects of exercise training in the antioxidant defense system reside intracellularly, including CuZnSOD, MnSOD, and CAT, EcSOD is the only one that functions extracellularly. An acute bout of endurance exercise promotes *EcSOD* gene transcription, and exercise training promotes EcSOD protein expression in skeletal muscle in mice [49,78,99,112,119], which could be recapitulated in culture myotubes following electrical stimulation [99]. Endurance exercise training has also been shown to increase the abundance of EcSOD in the aorta in mice [32,49] and in plasma in humans [106]. Importantly, exercise training increases EcSOD protein abundance in peripheral tissues, such as the heart, with no evidence of increased transcription of the EcSOD gene in these peripheral tissues [11]. Exercise-induced EcSOD expression seems to be specific for endurance exercise, not induced by resistance exercise [106]. Finally, genetically engineered mice with enhanced EcSOD expression in skeletal muscle showed increased EcSOD levels in the blood and all peripheral tissues and organs, such as the kidneys, liver, heart, lung, and adipose tissue [12]. These findings support that endurance exercise promotes EcSOD expression in skeletal muscle, the largest organ of the body, leading to enhanced extracellular antioxidant defense in the circulation and peripheral tissues as the molecular transducer of the benefits of exercise to health and disease (Fig. 1).

Several studies have investigated the signaling mechanism of exercise-induced EcSOD expression. In human aortic smooth muscle cells, the nitric oxide (NO) donor diethylenetriamine-NO (DETA-NO) increased EcSOD expression in a time- and dose-dependent manner, which appears to be dependent on cyclic GMP (cGMP)/protein kinase (PKG) and p38 mitogen-activated kinase (p38 MAPK) [32]. These findings were corroborated by treadmill exercise *in vivo* in mice lacking endothelial nitric oxide synthase (eNOS) [32]. The direct evidence of NO signaling in exercise-induced EcSOD expression in skeletal muscle came from the studies where DETA-NO treatment induces *EcSOD* mRNA and transcription in culture C2C12 myoblasts [116], and systemic administration of endogenous NO donor *S*-nitrosoglutathione (GSNO) results in increased EcSOD protein expression in skeletal muscle [78]. More recently, Yamada et al. presented evidence that exercise-induced EcSOD expression is dependent on nuclear factor erythroid 2-related factor 2 (Nrf2) in oxidative soleus muscle, but not glycolytic white vastus lateralis muscle [112]. The precise mechanism(s) that controls EcSOD expression in peripheral tissues in response to exercise training remain to be fully elucidated in future studies.

## 3 Catabolic muscle wasting

Cachexia is a clinical syndrome characterized by body weight loss, muscle and adipose tissue wasting and inflammation that is associated with many chronic disease conditions, such as cancer, sepsis, diabetes, COPD, CHF and renal failure. Oxidative stress has been shown to play a critical role in cardiac cachexia, and EcSOD activity and abundance are reduced in skeletal muscle in CHF [15,63]. On the contrary, it is well-known that slow-twitch, oxidative muscles are resistant to catabolic muscle wasting under chronic disease conditions [1,10,61,67,73,97]. These findings have been recapitulated in animal models [67,87,94]. For example, in a transgenic mouse model of CHF with cardiac-specific overexpression of calsequestrin, slow-twitch, oxidative muscle maintained muscle mass, whereas fast-twitch, glycolytic muscles underwent profound atrophy with sarcomere degeneration, loss of mitochondria and dramatic induction of the muscle atrophy F-box (*MAFbx*)/*Atrogin-*1 mRNA in the proteasome degradation system [67]. Even within the same muscle, glycolytic type IId/x and IIb fibers, but not oxidative type I and IIa fibers, displayed significantly decreased cross-sectional area [67].

**Fig. 2:**
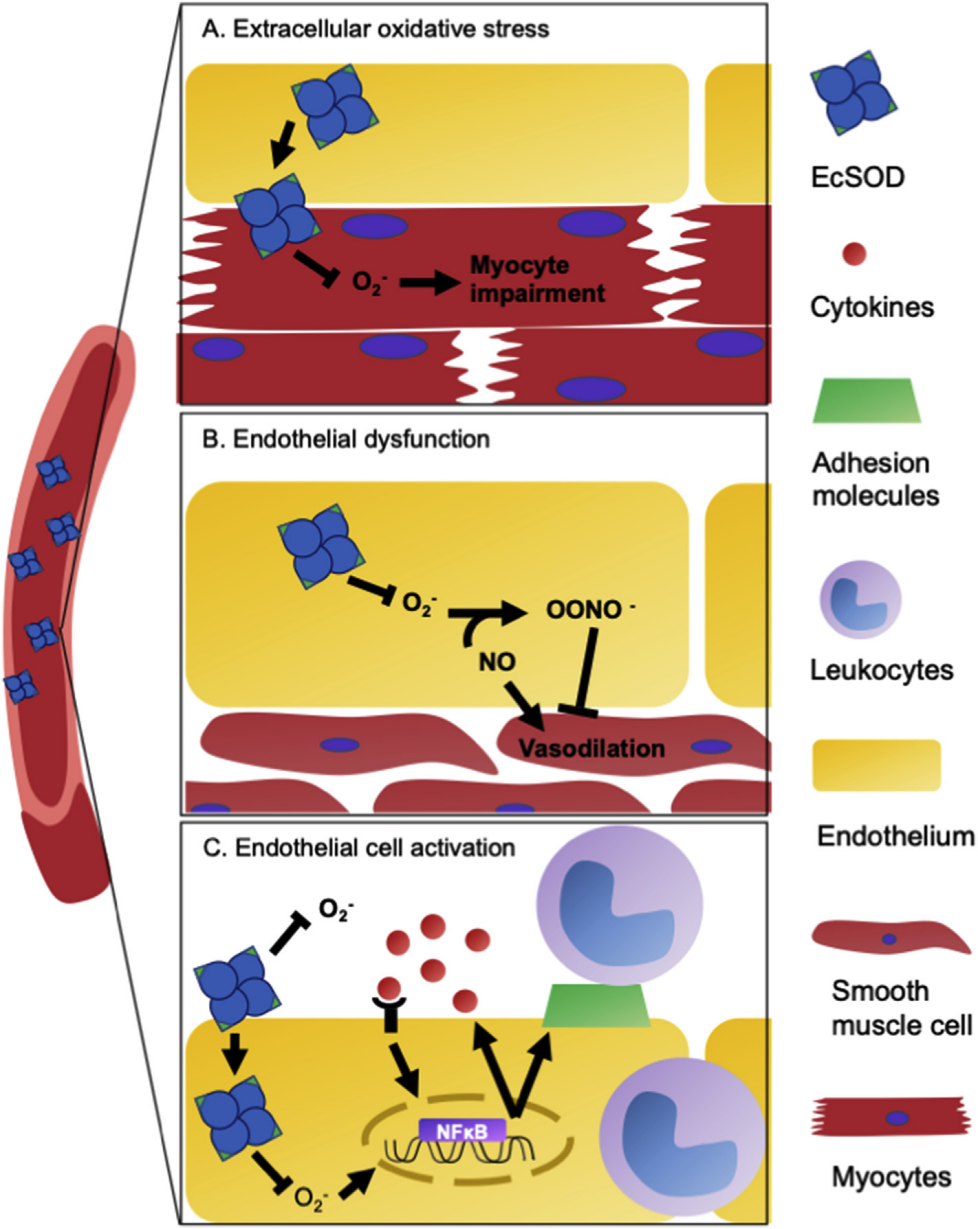
**Elevated EcSOD abundance/activity prevents extracellular oxidative stress endothelial dysfunction and endothelial cell activation by scavenging superoxide anion.** A) EcSOD originating from the skeletal muscle by exercise can be redistributed to nearby myofibers to the myocardium where it removes ROS and prevents myocyte impairment. B) Under normal conditions, NO generated in endothelial cells causes vasodilation in the adjacent smooth muscle cells in arteries. Increased superoxide reacts with nitric oxide (NO), produce peroxynitrite (OONO^−^), which inhibits vasodilation. Exercise-induced increase of EcSOD promotes removal of O_2_^.-^ and preserved NO availability to effectively prevent endothelial dysfunction. C) O_2_^.-^ stimulates pro-inflammatory cytokines and expression of cell surface adhesion molecules, such as VCAM-1, ICAM-1, and E-selectin. Cytokines recruits the leukocytes to interact with endothelial cells through the cell surface adhesion molecules, leading to a vicious cycle of endothelial cell activation as well as leukocyte transendothelial migration, causing tissue damage.

The molecular mechanism underlying the protection associated with oxidative phenotype appears to be NO-mediated enhancement of the antioxidant defense as systemic administration of endotoxin (lipopolysaccharide, LPS) to mimic sepsis led to induced inducible NO synthase (*iNOS*), *CuZnSOD*, *MnSOD*, *EcSOD* and *CAT* mRNAs in atrophy resistant oxidative muscle, but not in atrophy prone glycolytic muscle in mice [116]. Importantly, NO donors enhanced *iNOS* and *EcSOD* expression and blocked cytokine/endotoxin-induced *MAFbx/atrogin-1* expression in cultured myoblasts and in skeletal muscle *in vivo*, linking NO-mediated upregulation of EcSOD to the protection against catabolic muscle wasting in skeletal muscle [78,116]. Importantly, EcSOD is highly expressed in slow-twitch, oxidative muscles and induced by endurance exercise training and enriches at capillary endothelial cells in skeletal muscle in mice. Somatic gene transfer or transgenic overexpression of EcSOD provided potent protection against muscle atrophy induced by either glucocorticoidism or CHF and vascular rarefaction [78]. Therefore, exercise-induced EcSOD expression is sufficient to protect muscle fibers from catabolic wasting induced by intracellular oxidative stress in skeletal muscle (Fig. 2A). These findings are highly consistent with the findings that exercise training attenuates muscle wasting and prevents oxidative stress in CHF patients [21]. It remains to be elucidated if enhanced EcSOD expression functions intracellularly in the producing muscle fibers or extracellular on the surface of capillary endothelial cells and in the extracellular matrix in a paracrine manner.

## 4 Cardiovascular Disease

Cardiovascular disease encompasses a group of diseases impacting the heart and blood vessels, including hypertension, atherosclerosis, MI and CHF [83]. Cardiovascular disease continues to be the leading cause of death to Americans [8], and oxidative stress actively participates in each of these diseases.

### 4.1 Hypertension

Hypertension affects more than 40% of adults worldwide and is associated with stroke, MI, CHF, and other cardiovascular diseases [65]. Exercise training has been recommended by the American College of Sports Medicine and American Heart Association as a cornerstone of non-pharmacologic therapy for hypertension [86]. However, the underlying mechanism(s) of the benefit of exercise training in this regard are unclear.

Endothelium of resistance arteries is known to regulating blood pressure by altering vascular tone, cell growth and the interaction between the immune cells and the vessel wall. It also synthesizes growth factors and thrombo-regulatory molecules and responds to physical and chemical signals. Endothelial dysfunction is a condition of deteriorated endothelium-dependent vasodilatation that plays an important role in pathogenesis of hypertension and many other cardiovascular problems [48]. Therefore, endothelial dysfunction and EcSOD at endothelium are potential therapeutic targets for hypertension (Fig. 2B).

In a mouse model of hypertension induced by angiotensin II minipump implantation in mice, there were increases of vascular EcSOD expression as a potential compensatory mechanism to restore homeostasis of hemodynamics [33,64]. Experimental studies confirmed this hypothesis. Specifically, global EcSOD knockout mice (*EcSOD*^*−/−*^) showed similar blood pressure at baseline compared with the wild type littermates, but displayed exacerbated hypertension following angiotensin II administration [41]. A novel rat strain with loss-of-function mutation of *EcSOD*^*E124D*^ in the Dahl/Salt Sensitive (Dahl/SS) background developed age-dependent arterial hypertension along with kidney failure and cardiac hypertrophy [45]. Similarly, mice with smooth muscle specific knockout of the *EcSOD* gene developed age-dependent hypertension and aortic stiffening, supporting that vascular EcSOD is protective against hypertension [110].

In stroke-prone spontaneous hypertensive rat, adenovirus-mediated overexpression of EcSOD increased blood vessel and advential level of EcSOD and NO availability and reduced endothelial dysfunction, which could not be achieved by MnSOD [27]. Similarly, Chu et al. also found that adenovirus-mediated gene transfer of EcSOD, but not EcSOD(R123G) which lost binding to arteries and kidneys, resulted in reduced arterial pressure *in vivo* and restored vasoreactivity *in vitro* in spontaneously hypertensive rats (SHR). EcSOD, but not EcSOD(R213G), also reduced superoxide and nitrotyrosine in the aorta of SHR [17]. These finding support the notion that enhanced EcSOD expression from a different organ (liver) can exert profound antihypertension function dependent on the binding to the vasculature through HBD.

Taking advantage of *EcSOD*^*−/−*^ mice with intravenous administration of human recombinant EcSOD *in vivo*, Jung et al. determined the involvement of EcSOD in the control of blood pressure and endothelium-dependent responses in angiotensin II-induced hypertension and renovascular hypertension induced by the two-kidney, one-clip model. *EcSOD*^*−/−*^ mice had exacerbated hypertension, which were mitigated by recombinant EcSOD. *EcSOD*^*−/−*^ mice exhibited attenuated acetylcholine-induced vasodilation, which was further depressed in hypertension models along with increased vascular superoxide, and the protective role of EcSOD is NO-dependent [54]. These findings revealed the importance of endogenous EcSOD as an antagonistic principle to vascular superoxide and preserve NO bioavailability to prevent endothelial dysfunction (Fig. 2B), which is critical for control of blood pressure and vascular function in hypertension [55]. However, there is still no direct evidence that the superb efficacy of exercise training in treating hypertension is mediated by enhanced EcSOD expression from skeletal muscle and/or other tissues/organs.

### 4.2 Atherosclerosis

Atherosclerosis is a chronic, progressive vascular disease that involves inflammation response, oxidative stress, macrophage alteration, and inflammatory factor interaction, and endurance exercise has been demonstrated to be the most effective preventative intervention. Oxidative stress is known to modulate atherogenic dyslipidemia, and gene polymorphisms in EcSOD may contribute to oxidative stress-related lipid abnormalities and the development of atherosclerosis. *EcSOD rs2*536512-AA and *rs2*855262-CC variants have been linked to increased risk for hypertriglyceridemia with low high-density lipoprotein cholesterol level, which is known to be associated with coronary atherosclerosis [24]. Combined polymorphisms in antioxidant-related genes increased the risk of dyslipidemia related to atherosclerotic severity, predictive of atherogenic dyslipidemia. Furthermore, high levels of superoxide have been found in aortic valve and aorta in hypercholesterolemic mice and in older humans, suggesting a role of superoxide in atherogenesis [43,53].

Adenovirus-mediated overexpression of EcSOD in the adventitia of rat femoral arteries reduced neointimal formation with reduced oxidative stress, smooth muscle cell proliferation, iNOS expression, apoptosis and collagen content in the vascular wall following a cuff-injury [81], suggesting that enhanced EcSOD activity in the vascular wall is sufficient to protect against atherosclerosis. Interestingly, double knockout of the *EcSOD* and *ApoE* genes (*EcSOD*^*−/−*^*/ApoE*^*−/−*^) resulted in smaller lesions than *ApoE*^*−/−*^ mice, a mouse genetic model of atherosclerosis, and increased cholesterol, but serum or urine lipid peroxidation and oxLDL abundance in the aortic root suggesting that basal EcSOD may play a minor role in atherosclerosis [96], although this does not exclude the possibility that enhanced EcSOD abundance/activity in the vasculature is protective. Consistent with a role of EcSOD in exercise training-induced benefits in prevention of atherosclerosis, treadmill running in mice induced expression of *EcSOD* mRNA in skeletal muscle and aorta [49]. Importantly, voluntary wheel running in *ApoE*^*−/−*^ mice on normal chow diet led to a significant reduction in atherosclerotic lesion at the aortic root associated with reduced systemic inflammation [5]. These studies paved the way for future research to ascertain the role of exercise induced EcSOD in blood vessels or from skeletal muscle in protection against atherosclerosis.

### 4.3 Myocardial infarction (MI)

Myocardial infarction is mainly caused by atherosclerosis but directly related to oxidative stress during ischemic reperfusion. A T-allele of *rs2284659* variant in the promoter of the *EcSOD* gene with higher plasma EcSOD and lower plasma advanced oxidation protein products has been shown to be inversely associated with incidence of MI and mortality in diabetic patients [74]. This finding suggests a protective role of EcSOD in reducing the risk and severity of MI. On the contrary, advanced age patients with in-stent reocclusion had reduced plasma EcSOD, NO and eNOS and elevated oxidative stress markers, suggesting that reduced EcSOD may cause reocclusion in these patients [68].

Pre-emptive overexpression of EcSOD specifically to the myocardium reduced infarct size, improved ventricular function and survival in rat models of MI induced by ischemia/reperfusion [3,77]. AAV9-mediated, cardiac-selective gene expression via systemic administration elevated EcSOD enzyme activity in the heart and provided protection against both acute MI and subsequent left ventricle remodeling along with increased myocardial capillary fractional area and decreased neutrophil infiltration, making systemic administration of AAV9-mediated it a viable gene therapy for MI [60]. It is conceivable that exercise training-induced EcSOD levels in the myocardium as observed in mice following long-term voluntary wheel running [11] may prove to be effective in protection against MI.

### 4.4 Chronic heart failure (CHF)

CHF is a leading cause of morbidity and mortality due to an inability to pump enough blood throughout the body to maintain metabolic homeostasis in peripheral tissue [8,76]. Endothelium-bound EcSOD and xanthine-oxidase activities were found to be significantly reduced and increased, respectively, in patients with CHF accompanied by impaired flow-dependent, endothelial-mediated vasodilation [63]; these abnormalities in hemodynamics likely contribute to the development of cardiac dysfunction.

Consistent with the notion that EcSOD plays an important role in protecting the heart against oxidative stress in the development of cardiac hypertrophy, fibrosis, and contractile dysfunction, reduced EcSOD function in patients carrying R213G SNP is tightly associated with increased risk for these cardiovascular abnormalities. For example, EcSOD R213G SNP results in more than 2-fold increase in risk of cardiovascular disease and CHF in diabetes patients [59]. In a prospective, double-blinded, double-dummy study, either statin or statin plus xanthine-oxidase inhibitor treatment resulted improved flow-dependent endothelial-mediated vasodilation concurrent with increased EcSOD activity and decreased oxidative stress marker [42]. Importantly, exercise training has not only been shown to reduce the risk of CHF but also to be beneficial to patients already diagnosed with CHF [14].

Preclinical studies have shed lights on the role of EcSOD in CHF. EcSOD expression is decreased in MI-induced CHF [39]. MI-induced left ventricle hypertrophy, fibrosis and systolic dysfunction are exacerbated in *EcSOD*^*−/−*^ mice along with pronounced increase of nitrotyrosine and impairment of intracellular signaling [105]. In a model of pressure overload-induced CHF, *EcSOD*^*−/−*^ mice developed more severe left ventricular hypertrophy, fibrosis, dilation, and greater reduction of fractional shortening and rate of pressure development along with pulmonary congestion. These findings were accompanied by significant oxidative stress [69]. Conversely, in a rat model of CHF, intravenous administration of adenovirus carrying EcSOD, not EcSOD with deleted HBD or R213G mutation, resulted in increased EcSOD binding to endothelium and increased SOD activity in the aorta, reduced levels of superoxide and peroxinitrite, and improved relaxation to acetylcholine and ADP in the aorta and mesenteric artery [51]. These findings all suggest a critical role of EcSOD in protecting heart against CHF.

More promising evidence came from exercise studies in mice. Endurance exercise trained mice had increased EcSOD levels in the serum and heart without increasing EcSOD gene expression in the heart [11]. Genetically engineered mice with skeletal muscle-specific overexpression of EcSOD to recapitulate the effects of exercise training showed significantly reduced diabetic cardiomyopathy with reduced cardiac hypertrophy, fibrosis, oxidative damage as well as reduce impairment of cell signaling in a model of late stage type 1 diabetes induced streptozotocin (STZ) injection [11]. The findings strongly support that EcSOD produced in skeletal muscle, which is enhanced by endurance exercise, is sufficient to protect cardiac function and reduce intracellular oxidative stress and damage to the heart under the condition of severe diabetes [11].

## 5 Pulmonary disease

Oxidative stress in the lung tissue participates in the pathogenesis of various pulmonary pathologies, including COPD and ALI/ARDS. EcSOD is highly expressed in lungs specifically located in the extracellular matrix, the junctions of airway epithelial cells, airway smooth muscle and the endothelium of vessels of the lung [58], scavenging superoxide in this tissue in our body that directly deals with oxygen in the ambient air. Accumulating evidence shows that EcSOD polymorphisms are associated with a decline in lung function in rodents and humans, and altered susceptibility to lung injury as a result of alteration of EcSOD level and function in the lung tissue [7,22,36].

### 5.1 ALI/ARDS

ALI/ARDS is a disease condition of respiratory failure with a mortality rate of about 40% and accounts for approximately 75,000 annual deaths in the U.S [92,109]. General antioxidants have failed in clinical trials due to lack of targeting specificity [101]. The innate immune response plays a profound role in the pathogenesis of ALI/ARDS, which involves cascades of events with neutrophils, macrophages, and dendritic cells partaking in mediating lung injury. A key step in ALI/ARDS is the dysregulation and recruitment of activated neutrophils to the lung microvasculature, interstitial and alveolar space [85]. Excessive neutrophil activation and accumulation leads to increased reactive oxygen species (ROS) and pro-inflammatory mediators, resulting in acute lung injury [84,85]. Therefore, neutrophil adhesion and subsequent activation by activating endothelium, a condition called endothelial cell activation, is an essential, early step in the inflammatory pathogenesis of ALI/ARDS [118]. The degree of endothelial cell activation and lung injury is strongly associated with the outcomes in ALI/ARDS [108] (Fig. 2C).

In line with the notion that EcSOD plays a critical role in the first line of defense against superoxide generation in the lung tissue, acute loss of EcSOD in adult mice (inducible *EcSOD*^*−/−*^ mice) causes a dramatic increase of mortality with ALI/ARDS even in ambient oxygen although global *EcSOD*^*−/−*^ mice are viable and tolerate exposure to ambient oxygen levels without difficulties. This severe pathological condition could be rescued by intranasal administration of EcSOD mimetic [40]. In contrast, low mortality rates were observed in EcSOD-overexpressing animals exposed to hyperoxia [29,40].

Exercise training and therapeutic exercise has been shown to limit alveolar neutrophilia through modulation of systemic neutrophil chemokines in lung-injured mice and humans [28,91]. In a mouse model of endotoxemia induced by LPS, enhanced EcSOD expression in skeletal muscle profoundly protected the mice from developing ALI/ARDS with reduced mortality while *EcSOD*^*R213G*^ mice are more vulnerable [12]. Importantly, the elevated EcSOD in the blood has been shown to be sufficient to inhibit endothelial cell activation and inflammatory cell adhesion induced by endotoxemia [12] (Fig. 2C). These findings strongly support that enhanced EcSOD expression from skeletal muscle or other tissues/organ, which can be redistributed to the lung tissue, could be a viable preventative/therapeutic measures in reducing the risk and severity of ALI/ARDS. Considering the current outbreak of the 2019 Novel Corona virus infection (COVID-19), which is an infectious disease that leads to progressive ALI/ARDS in many patients [6,103,107,113], it is conceivable that regular exercise might be effective in preventing while EcSOD gene/protein therapy might be effective in treating ALI/ARDS under the condition of COVID-19 infection.

### 5.2 COPD

Oxidative stress in the lung tissue is at the center of the pathogenesis of COPD [71]. In clinical studies, *rs8192287* (E1) and *rs8192288* (I1) SNPs are tightly coupled and associated with reduced lung function [22,100] and elevated risk of COPD [22], whereas *R213G* SNP is associated with resistance to COPD [57,98,115]. These findings were somewhat paradoxical at the first glance, but careful research reveal that EcSOD plays an important role in the pathogenesis of COPD with disparate effects depending on its tissue distribution. The former two SNPs simply lead to reduced expression of EcSOD in the lungs [22], whereas *R213G* SNP decreases the matrix binding affinity without altering enzyme activity but lead to redistribution of active EcSOD into the plasma and epithelial lining fluid [47]. A major difference between the *R213G* SNP and the E1 and I1 SNPs is the increased diffusion of EcSOD into the alveolar lining fluid of the lung, which serve as an antioxidant shield that protects the lungs against oxidants. Consistent with this notion is the finding that EcSOD R213G polymorphism is associated with reduced risk of COPD for smokers, but not for non-smoker [57,115]. Gaurav et al. have also reported that R213G SNP carriers in humans have higher EcSOD in airway lining fluid and reduced allergic airway inflammation with reduced asthma-related symptoms in allergic asthma, which were recapitulated in *EcSOD*^*R213G*^ mice [38].

Animal studies with genetic manipulation to mimic human SNPs have shed light on the role of EcSOD in the pathogenesis of COPD. Ganguly et al. showed correlation between EcSOD polymorphisms, lung EcSOD expression, and pulmonary function in mice. JF1/Msf mice, known with decreased ventilation efficiency, were found to have 3 EcSOD promoter SNPs and decreased EcSOD transcript, protein expression, and activity in the lungs compared to C3H/HeJ mice [36]. This study illustrates correlation of pulmonary expression of EcSOD with decreased lung function and increased susceptibility to COPD. Interestingly, *EcSOD*^*R213G*^ knock-in mice exhibited enhanced resolution of inflammation with subsequent protection against fibrosis following bleomycin treatment compared with wild type littermates [75], which was later confirmed by RNAseq analysis showing induction of inflammatory and immune response genes were suppressed in *EcSOD*^*R213G*^ mice [37]. Similar findings were observed for models of allergic airway inflammation [38,47]. These findings suggest that the redistributed EC-SOD due to the R213G SNP attenuates the dysregulated inflammatory responses. These findings are amazingly consistent with the findings in R213G carriers in humans [57,115]. We speculate that endurance exercise may promote EcSOD expression in skeletal muscle, which could be redistributed to peripheral tissues, such as lung, providing protection against airway inflammatory disease and COPD.

## 6 Renal disease

Renal injury and oxidative stress are common in a variety of renal diseases, including chronic kidney disease (CKD) and acute kidney injury (AKI). A protective role of EcSOD in renal disease has been implicated, but its relationship to exercise training is poorly understood.

### 6.1 Acute kidney injury (AKI)

Mouse models in which the *EcSOD* gene is mutated or SOD inhibitors are used suggest protective role of EcSOD against renal injury [19,45,95,104]. *EcSOD*^*−/−*^ mice were more susceptible to histological damage, oxidative stress and increased serum creatinine caused by adverse drug reaction [104]. Similarly, when SOD inhibitor was used in a rat sepsis model induced by cecal ligation-puncture (CLP), renal blood flow decreased and nitrotyrosine content increased 48 h post CLP [19]. EcSOD has also been shown to be protective against renal ischemia/reperfusion injury as demonstrated by increased nitrotyrosine and increased renal cast formation 24 h post ischemia/reperfusion in *EcSOD*^*−/−*^ compared with wild type mice [95]. Finally, when LPS was used to induce endotoxemia, mice with muscle specific transgenic overexpression of EcSOD had reduced blood markers of kidney injury in blood urea nitrogen (BUN) and serum creatinine (SCR) [12]. Further, intraperitoneal injection of serum carrying 6-fold more EcSOD from these transgenic mice reduced LPS-induced kidney injury in wild type recipient mice [12]. Interestingly, in a heterogenic parabiosis model in which a wild type mouse was surgically fused to and shared blood with a muscle specific transgenic EcSOD mouse, the wild type mice were protected from LPS induced kidney injury compared with parabiosis of two wild type mice [12]. Importantly, *EcSOD*^*R213G*^ mice had greater kidney injury along with more profound endothelial cell activation following LPS-induce endotoxemia, supporting that systemic administration of EcSOD is sufficient to protect against kidney injury [12]. More importantly, EcSOD derived from muscle, which could be promoted by exercise training, is sufficient for this protection.

### 6.2 Chronic kidney disease (CKD)

EcSOD activity has been shown to be reduced in patients with CDK and animal models of CKD [104]. In type 2 diabetes mellitus patients with albuminuria, a hallmark of kidney disease, EcSOD activity is reduced [26]. As albuminuria becomes more severe from normoalbuminuria, microalbuminuria to finally macroalbuminuria, EcSOD activity declines, providing strong association of decreased EcSOD activity with increased renal injury. As albuminuria became more severe, BUN and SCR increased [26]. When EcSOD was mutated at amino acid 124 from glutamic acid to aspartic acid (*EcSOD*^*E124D*^) in rats, spontaneous CKD occurred in an age-dependent manner as a result of increased renal oxidative stress, inflammation, and fibrosis [45]. Additional animal studies provided more definitive evidence for the protective role of EcSOD against CKD. In a model of diabetic nephropathy in rats induced by STZ-injection, Kuo et al. showed that four weeks of treatment with human recombinant EcSOD improved lifespan and decreased kidney damage [62], demonstrating the protective function of EcSOD against CKD. Future research should investigate the potential benefits of endurance exercise training in CKD.

## 7 Multiple Organ Dysfunction Syndrome (MODS)

MODS, a major contributor of intensive care unit (ICU) mortality, occurs when multiple organs are damaged to the point where they cannot maintain homeostatic function. Emerging evidence suggests that oxidative stress-induced endothelial cell activation (induced expression of adhesion molecules) in vital organs is a key step in the pathology as it leads to exacerbated inflammatory cell infiltration and tissue damage [4]. Endothelial cell activation in the vasculature can further stimulate immune cell-endothelial cell interaction and production of free radicals, contribute to a vicious cycle in the development of MODS. Given the role of ROS in inducing endothelial cell activation and general oxidative stress in MODS, general antioxidants have been investigated in protection against MODS in human. Unfortunately, none of the general antioxidants, which have no target specificity, have all failed in these clinical trials [101]. Through parabiosis experiments and intraperitoneal serum transfusions, we demonstrated that EcSOD protected against LPS-induced MODS by preserving lung function, reducing endothelial cell activation, and decreasing endothelial cell adhesion [12]. Similarly, in a sepsis model, administration of an exogenous SOD mimetic protected the lung by reducing oxidative damage and inflammation [20]. Together these studies suggest boosting EcSOD abundance and activity may protect against MODS, particularly by enhanced expression of EcSOD from a remote organ like skeletal muscle. Serum carrying elevated EcSOD protein from skeletal muscle was shown to be sufficient to reduced endothelial cell activation with suppressed VCAM-1 expression *in vitro* and *in vivo* [12] (Fig. 2C). Intravital imaging of inflammatory cell-endothelial cell interaction revealed reduced inflammatory adhesion conferred by EcSOD protection in peripheral tissue vasculature under the condition of endotoxemia in muscle-specific EcSOD transgenic mice [12].

## 8 Other disease

Exercise-induced enhancement of EcSOD expression has also been confirmed or implicated in protection against variety of other disease conditions. Osteoarthritis (OA), often presented as increased mechanical damage to joint cartilage, is associated with decreased EcSOD levels in the joint fluid and cartilage in OA patients [90]. In STR/ort mice, a mouse model of OA, EcSOD deficiency was observed prior to evidence of OA, suggesting that oxidative damage and EcSOD deficiency play a role in the onset of OA [90]. We have shown that high-fat diet-induced obesity in mice caused OA and systemic inflammation, which was mitigated by voluntary wheel running [44]. The protective effect of endurance exercise is likely mediated by EcSOD in the cartilage tissues. In another study in mice, long-term forced treadmill running improved cartilage histology in the joint in wild type mice but this beneficial effect with running was lost in heterozygous *EcSOD*^*R213G*^ knock-in mice, suggesting that EcSOD, specifically EcSOD binding to the cartilage through the HBD, is required for the benefit of exercise to the joint [82]. Further, *EcSOD*^*R213G*^ runner mice had less robust increase in bone strength compared with wild type runner mice. Therefore, exercise-induced EcSOD plays an important role in protecting cartilage from mechanical load-induced damage [82].

EcSOD is also important in retinopathy as reduced serum EcSOD is a risk factor for diabetic retinopathy (DR) [120]. As DR progresses, abnormal neovascularization occurs and serum EcSOD gradually declines, suggesting an association between EcSOD and progressive DR. Others too have proposed that EcSOD may be targeted a potential therapeutic to protect against oxidative stress and DR [52].

## 9 Conclusions

EcSOD scavenges superoxide as the first line of defense in extracellular space. Numerous human and animal studies have investigated the role of EcSOD in various pathological conditions and demonstrate the deleterious effects of reduced EcSOD levels and activities in disease development as well as the protective effects of enhanced EcSOD in reducing ROS and oxidative damage under disease conditions (Fig. 1). The facts that EcSOD expression is promoted by endurance exercise in skeletal muscle and can be redistributed to other vital tissues to protect the target tissues against oxidative damage in various pathological processes (Fig. 2) strongly support exercise-induced EcSOD as an effective therapeutic intervention for prevention and treatment of numerous oxidative stress-related diseases.

## Declaration of competing interest

The authors have no conflict of interest to declare.
